# A Rare Case Report on Dedifferentiated Liposarcoma of the Thigh Mimicking an Abscess With Vascular Features: A Diagnostic Challenge With Fatal Outcome

**DOI:** 10.1002/ccr3.73630

**Published:** 2026-09-25

**Authors:** Rashid Shahriar Sazal, Md. Hashibul Hasan Shawon, Mahin Zubaer, Md. Abul Kalam Azad, Khaled Mahbub Murshed, Kazi Ali Aftab

**Affiliations:** ^1^ Department of Internal Medicine Bangladesh Medical University Dhaka Bangladesh

**Keywords:** abscess mimic, dedifferentiated liposarcoma, diagnostic challenge, soft tissue sarcoma, sterile necrotic aspirate, thigh tumor

## Abstract

Dedifferentiated liposarcoma (DDLPS) is an aggressive malignant soft tissue tumor comprising approximately 15%–20% of all liposarcomas. When presenting in the extremities, its atypical clinical and radiological features may mimic vascular or infectious conditions, posing a serious diagnostic challenge that can delay curative intervention. A 60‐year‐old diabetic female presented with a 6‐month progressive left thigh mass accompanied by intermittent fever, significant weight loss, and refractory anemia. Doppler ultrasonography initially suggested a hemangioma; contrast‐enhanced CT was interpreted as an organized abscess. Ultrasound‐guided aspiration yielded 600 mL of thick necrotic aspirate—Gram stain demonstrated no organisms, culture was sterile, and aspirate cytology showed RBC 98,000 and WBC 25,000, confirming the absence of pyogenic infection. Concurrent core biopsy from the lesion wall established the diagnosis of DDLPS (FNCLCC Grade 2). PET‐CT confirmed locoregional disease without distant metastasis. Following surgical referral, the patient died suddenly overnight before planned excision. To our knowledge, this is the first reported case demonstrating the combined triad of Doppler‐based vascular mimicry, CT‐based abscess diagnosis, and large‐volume sterile necrotic aspirate in extremity DDLPS, culminating in sudden pre‐operative death. This case highlights that histopathological evaluation—including concurrent biopsy of any aspirated deep soft tissue mass—must not be deferred regardless of apparent microbiological findings.

## Key Clinical Message

Atypical deep soft tissue masses mimicking abscess or vascular lesions should undergo early biopsy, especially when microbiological studies are negative. Large‐volume necrotic aspirate does not exclude malignancy, and concurrent histopathological evaluation is essential to avoid diagnostic delay.

**Abbreviations**
CDK4: cyclin‐dependent kinase 4
CECT: contrast‐enhanced CT
CT: computed tomography
DDLPS: dedifferentiated liposarcoma
FDG: fluorodeoxyglucose
FNCLCC: Fédération Nationale des Centers de Lutte Contre le Cancer
MDM2: murine double minute 2
MRI: magnetic resonance imaging
PET‐CT: positron emission tomography‐CT
WDL: well‐differentiated liposarcoma

## 1 Introduction

Liposarcoma (LPS) is the most common soft tissue sarcoma in adults, accounting for approximately 20% of all sarcomas [1, 2]. The current World Health Organization classification recognizes four principal histological subtypes: well‐differentiated (WDL), dedifferentiated (DDLPS), myxoid, and pleomorphic [3]. DDLPS—defined by non‐lipogenic sarcomatous transformation of a WDL component—comprises 15%–20% of LPS cases and carries substantially higher morbidity and mortality than WDL [4]. At the molecular level, co‐amplification of MDM2 and CDK4 on chromosome 12q13‐15 constitutes the defining molecular hallmark of WDL/DDLPS: MDM2 suppresses p53‐mediated apoptosis, while CDK4 drives unrestrained G1/S cell‐cycle progression [5].

Although DDLPS predominates in the retroperitoneum (> 80% of cases), extremity involvement—particularly in the thigh—does occur and is associated with unique diagnostic challenges [6]. Deep soft tissue location and extensive intra‐tumoral necrosis may render the radiological appearance indistinguishable from an infectious abscess or vascular malformation, causing clinically consequential delays in diagnosis [7, 8]. We present a case in which extremity DDLPS sequentially mimicked a hemangioma and then an abscess—yielding a large‐volume sterile necrotic aspirate mimicking pus—before histopathological confirmation, followed by sudden pre‐operative death.

## 2 Case Presentation

### 2.1 Patient Profile and Initial Evaluation

A 60‐year‐old female with a 10‐year history of type 2 diabetes mellitus, hypertension, hypothyroidism, and chronic kidney disease (CKD) presented with progressive generalized weakness (2 months), intermittent fever (1 month), and exertional dyspnea (7 days), alongside anorexia and involuntary weight loss of approximately 6 kg over the preceding month. Six months prior, a mildly painful postero‐medial left thigh swelling had been evaluated with Doppler ultrasonography, which demonstrated a 9.5 × 6.1 cm lesion with predominantly arterial flow, interpreted as a hemangioma, and managed conservatively without tissue sampling. But the size of the lesion was increasing gradually, prompting her to seek medical evaluation. The complete chronological sequence of clinical events is presented in Table 1.

**TABLE 1:** Clinical timeline from initial presentation to fatal outcome.

Timepoint	Event	Key finding/action
6 months pre‐admission	Initial thigh swelling	Doppler USG: 9.5 × 6.1 cm lesion with arterial flow → diagnosed as hemangioma; managed conservatively; no biopsy taken
Months 2–5	Progressive enlargement	Gradual increase in swelling; onset of systemic symptoms: fever, weight loss (~6 kg in 1 month), anorexia, weakness
Admission—Day 0	Hospital admission	Deep, tense, tender thigh swelling; unilateral leg edema to knee; moderate anemia; neutrophilic leukocytosis; elevated ESR/CRP; hypercellular marrow with myeloid hyperplasia
Day 1	CECT of thigh	~19 × 12 cm mixed‐density lesion; large central necrotic zone; irregular enhancing wall → interpreted as organized abscess
Day 3	MRI left thigh	18.4 × 11.2 × 10.6 cm encapsulated mass; T1 hypointense, T2/STIR hyperintense; peripheral enhancement; central necrosis; flow voids; adductor magnus involvement
Day 4	USG‐guided aspiration + core biopsy	600 mL thick necrotic aspirate (Gram stain: no organisms; C&S: sterile; cytology: RBC 98,000/WBC 25,000). Concurrent core biopsies from lesion wall obtained
Day 6	Second aspiration	Additional 150 mL thick necrotic aspirate drained
Day 8	Histopathology result	Spindle cell neoplasm; nuclear pleomorphism; atypical mitoses; lipoblastic differentiation; tumor necrosis → DDLPS FNCLCC Grade 2 confirmed
Day 10	PET‐CT (staging)	117 × 119 × 150 mm mass; peripheral FDG uptake; central necrosis; reactive marrow uptake; no distant metastasis
Day 11	Surgical referral	Referred for wide local excision following multidisciplinary oncological evaluation
Night—Day 11/12	Fatal outcome	Patient found unresponsive. ACLS initiated immediately; unsuccessful. Death confirmed. Autopsy not performed

Abbreviations: ACLS, advanced cardiac life support; C&S, culture and sensitivity; CECT, contrast‐enhanced computed tomography; MRI, magnetic resonance imaging; PET‐CT, positron emission tomography‐computed tomography; USG, ultrasonography.

### 2.2 Examination and Laboratory Investigations

Physical examination revealed a deep‐seated, tense, tender, warm swelling over the postero‐medial left middle thigh with intact overlying skin, and unilateral pitting oedema from the ankle to the knee. Moderate pallor was noted. Laboratory investigations demonstrated neutrophilic leukocytosis, markedly elevated ESR and CRP, moderate normocytic anemia refractory to erythropoiesis‐stimulating agents (necessitating multiple packed red cell transfusions), and deranged renal parameters consistent with CKD. Bone marrow examination showed hypercellular marrow with myeloid hyperplasia, attributed to chronic systemic inflammation. This biochemical and hematological constellation strongly suggested an infective etiology, confounding early clinical assessment.

### 2.3 Imaging Findings and Imaging–Pathology Correlation

**FIGURE 1:**
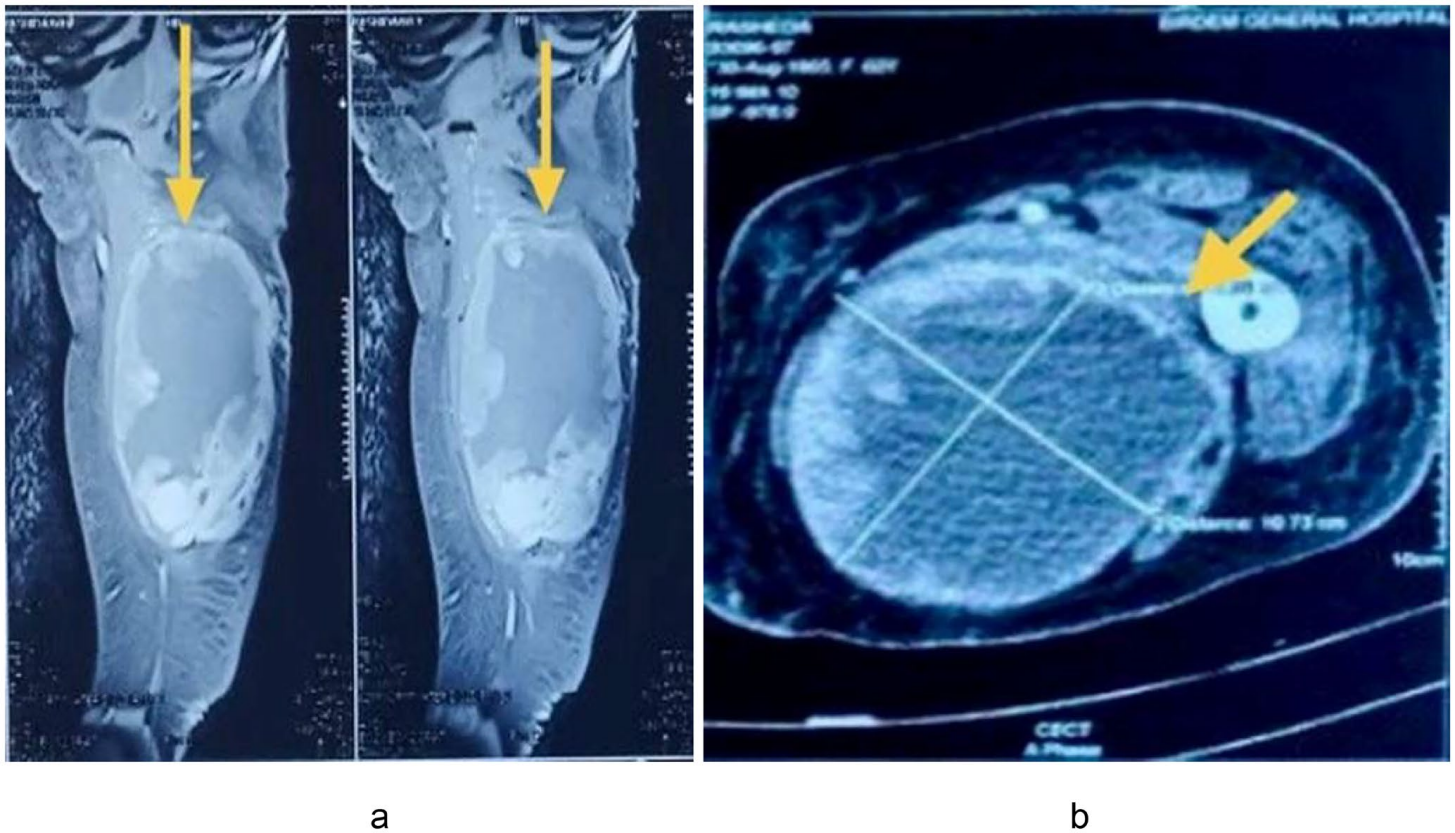
CECT scan of left thigh showing mixed density lesion having irregular thick wall soft tissue density area, containing large central necrotic component with irregular enhancing wall is seen in the muscles of the medial compartment of left thigh (yellow arrow in both a & b).

**FIGURE 2:**
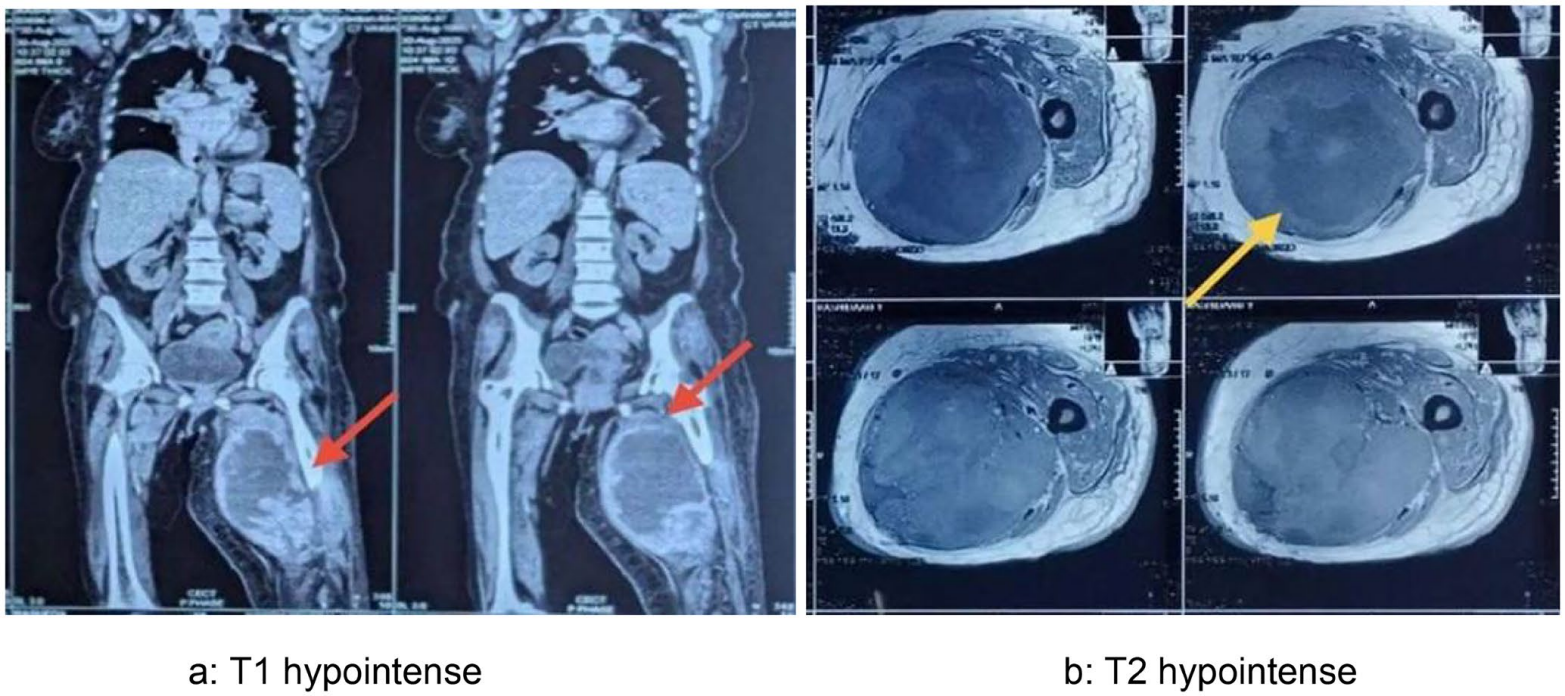
MRI of left thigh showing a large encapsulated mixed intensity lesion having flow void and central necrosis is noted in the postero‐medial aspect of left thigh which involves upper 2/3rd of the thigh. (red arrow in a and yellow arrow in b)

CECT of the left thigh (Figure 1a,b) revealed a large mixed‐density lesion (~19 × 12 cm) within the medial compartment muscles with a large central necrotic zone and an irregularly enhancing peripheral wall, initially interpreted as an organized abscess. MRI (Figure 2a,b) demonstrated an encapsulated mass (18.4 × 11.2 × 10.6 cm) with T1 hypo‐intensity, T2/STIR hyperintensity, peripheral enhancement, central necrosis, and prominent intra‐tumoral flow voids—the latter reflecting tumor‐associated angiogenesis rather than a true vascular malformation, and representing a critical imaging red flag for high‐grade malignancy. Muscle invasion of the adductor magnus was observed, extending through the upper two‐thirds of the thigh. Notably, the central necrosis visualized on CT and MRI corresponded directly to the large volume of acellular necrotic material obtained at subsequent aspiration, underscoring the direct pathological correlation between intra‐tumoral necrosis and its clinical mimicry of pyogenic collections.

### 2.4 Diagnostic Procedures and Histopathology

**FIGURE 3:**
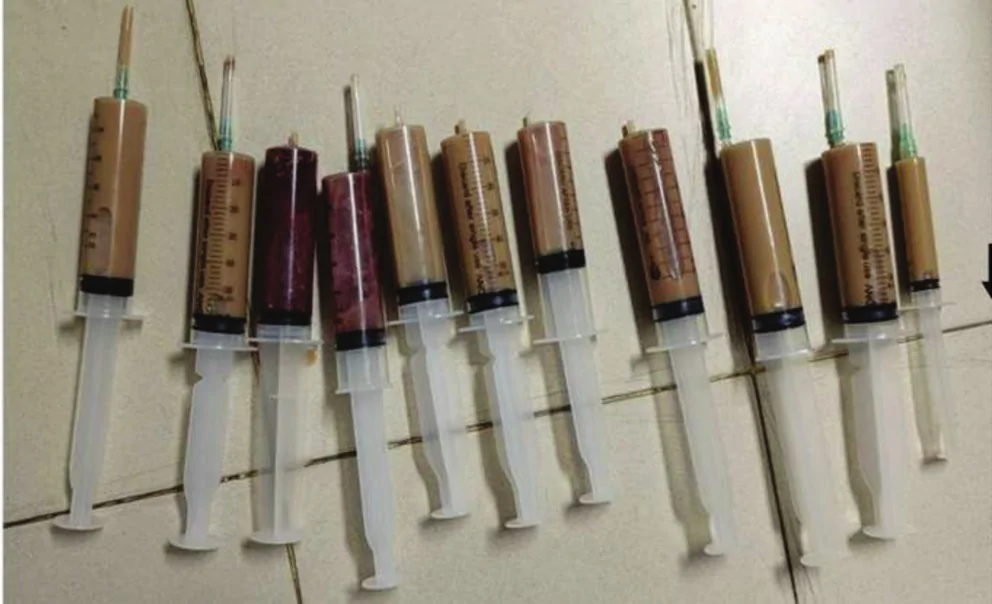
Purulent appearing necrotic fluid aspirated from left thigh mass.

Based on the radiological impression of an organized abscess, ultrasound‐guided aspiration was performed. This yielded 600 mL of thick, purulent‐appearing necrotic fluid (Figure 3). Gram stain of the aspirate demonstrated no organisms; culture and sensitivity (C&S) returned sterile; and aspirate cytology showed RBC 98,000 and WBC 25,000—a mixed cellular pattern atypical for pyogenic infection. These microbiological results reclassify this material as thick necrotic aspirate mimicking pus, confirming the absence of infection. An additional 150 mL was drained 2 days later. Concurrent multiple core biopsies were obtained from the lesion wall during the procedure.

**FIGURE 4:**
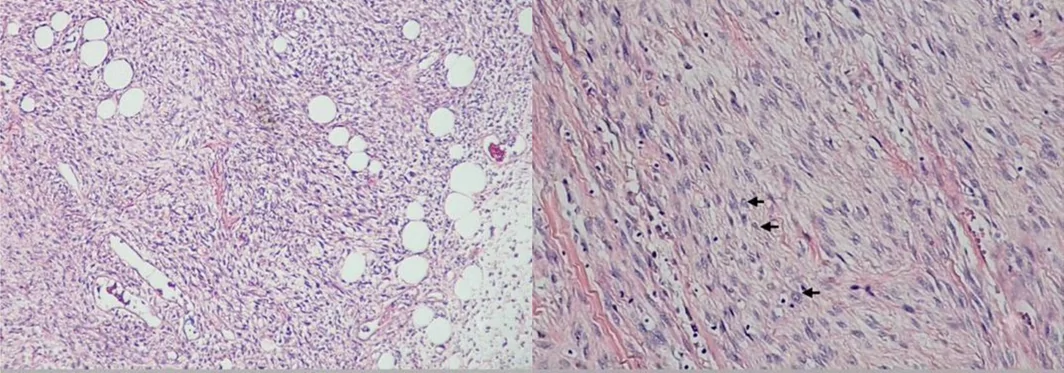
H&E‐stained photomicrographs showing lipomatous differentiation with a spindle‐cell proliferation (black arrow) and nuclear pleomorphism, consistent with the reported diagnosis of dedifferentiated liposarcoma.

Histopathological examination of the core biopsies revealed a spindle cell neoplasm with marked nuclear pleomorphism, atypical mitotic figures, lipoblastic differentiation, a well‐differentiated lipomatous component and extensive tumor necrosis, classified as DDLPS FNCLCC Grade 2 (Figure 4). MDM2/CDK4 immunohistochemistry and FISH were not performed due to resource constraints; however, the diagnosis was based on well‐established classical histomorphological criteria for DDLPS [9, 10].

### 2.5 Staging and Fatal Outcome

**FIGURE 5:**
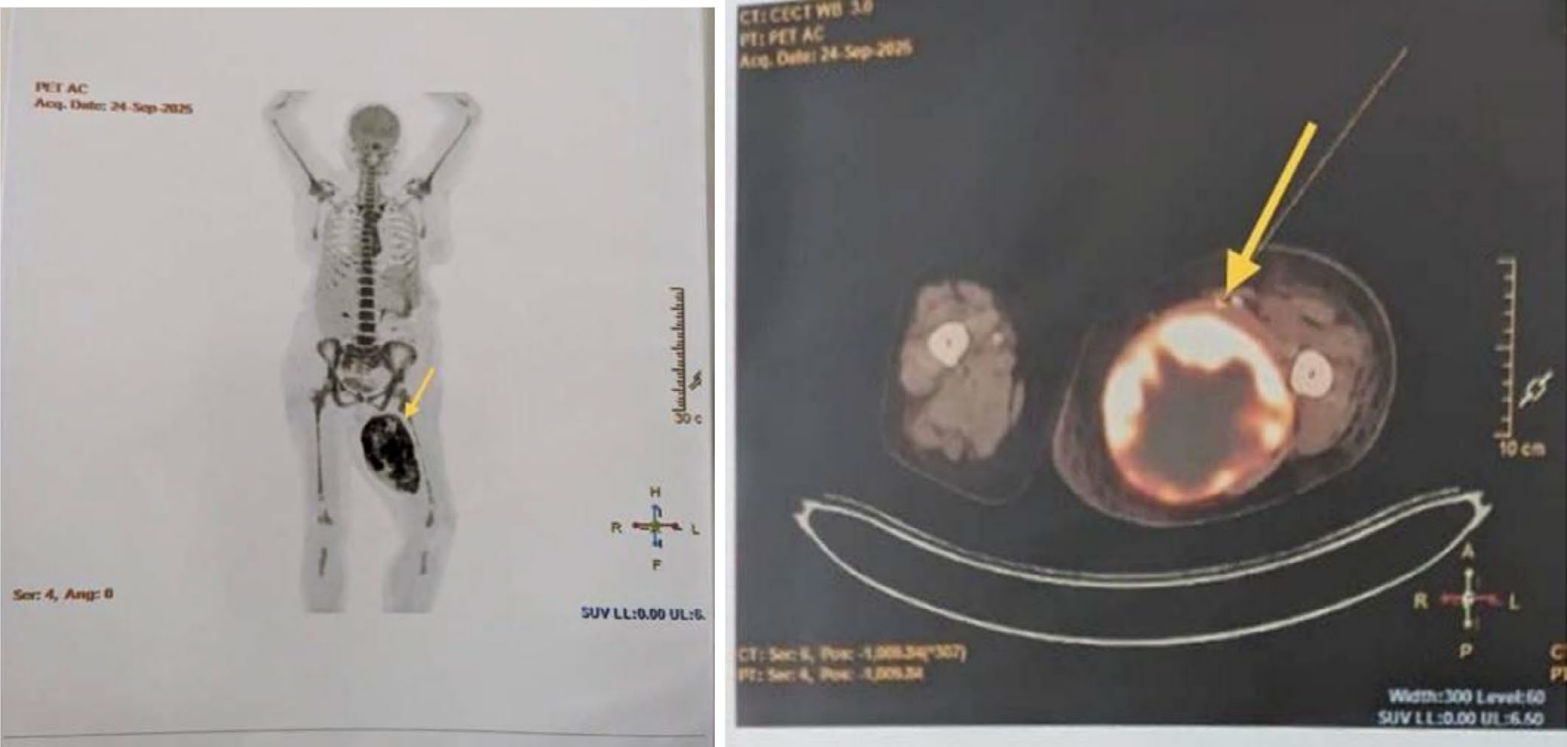
PET CT scan showing heterogenous density mass with peripheral FDG uptake having central necrotic area involving muscles of medial compartment of left thigh; consistent with biopsy proven sarcoma.

PET‐CT (Figure 5) demonstrated a metabolically active mass (150 × 119 × 117 mm) with peripheral FDG uptake and central necrosis, diffuse reactive marrow uptake, and no evidence of distant metastasis. Following multidisciplinary oncological evaluation, the patient was referred for wide local excision. That same night, she was found unresponsive in her hospital bed. Immediate advanced cardiac life support was initiated but was unsuccessful. Death was confirmed. Autopsy was not performed, and the formal cause of death could not be established.

## 3 Discussion

This case illustrates a diagnostically treacherous presentation of extremity DDLPS—a thigh mass that sequentially mimicked a hemangioma and a pyogenic abscess, yielding a large‐volume sterile necrotic aspirate before histopathological confirmation, followed by sudden pre‐operative death. Each element of this diagnostic sequence carries educational relevance for clinicians managing atypical deep soft tissue lesions.

### 3.1 Epidemiology

DDLPS represents approximately 15%–20% of all LPS and arises de novo in approximately 90% of cases [3]. In a retrospective cohort of 259 patients, the retroperitoneum was the primary site in 47.5%, while extremity involvement is comparatively less common [8]. A Korean multi‐center study of 107 extremity DDLPS patients reported 5‐year local recurrence‐free and disease‐specific survival rates of 84.7% and 87.8%, respectively—emphasizing that survival is critically dependent on achieving complete surgical excision [11]. Median age at DDLPS diagnosis is approximately 61–64 years, consistent with the present patient [8, 11].

### 3.2 Molecular Biology

The molecular signature of DDLPS—co‐amplification of MDM2 and CDK4 on chromosome 12q13‐15—suppresses p53‐mediated apoptosis and deregulates the G1/S cell‐cycle checkpoint, respectively [5]. MDM2 immunohistochemistry has a reported sensitivity of 100% for WDL/DDLPS, with CDK4 approaching 83%; FISH for MDM2 amplification is the most specific confirmatory modality [9]. In this case, molecular testing was not performed due to resource constraints, and diagnosis rested on classical FNCLCC Grade 2 histomorphology. Molecular confirmation should be obtained whenever feasible to maximize diagnostic certainty, particularly in atypical presentations [10].

### 3.3 Diagnostic Evolution

The misdiagnosis sequence in this case is explicable by tumor‐biological mechanisms. The prominent arterial Doppler signals reflected tumor‐associated neo‐angiogenesis—a hallmark of high‐grade sarcomas—rather than a vascular malformation. On CT, the large central necrotic zone with irregular peripheral enhancement closely simulated an organized abscess. MRI flow voids, attributable to pathological tumor angiogenesis, should in retrospect have prompted consideration of a high‐grade malignancy rather than infection.

The most diagnostically treacherous feature was the aspiration of 600 mL of purulent‐appearing necrotic fluid. In high‐grade sarcomas, rapid tumor growth outpacing vascular supply generates extensive intra‐tumoral hypoxia, coagulative necrosis, and liquefaction—yielding macroscopically purulent‐appearing material that is microbiologically sterile [12]. The negative Gram stain, sterile C&S, and atypical aspirate cytology (RBC 98,000; WBC 25,000) confirmed the absence of infection, reclassifying this material as necrotic tumor debris mimicking pus. The absence of clinical improvement with antibiotics was an additional red flag. Table 2 presents a structured comparison of features differentiating pyogenic abscess from DDLPS in this case.

**TABLE 2:** Differentiation between pyogenic abscess and DDLPS in the present case.

Feature	Typical pyogenic abscess	DDLPS (this case)	Clinical implication
Lesion size	Usually < 5 cm	Massive—19 × 12 cm	Red flag
Onset	Acute (days–weeks)	Insidious—6‐month history	Red flag
Gram stain	Organisms present	Negative—no organisms	Critical differentiator
Culture and sensitivity	Positive	Sterile	Critical differentiator
Aspirate cytology	Neutrophil predominance	RBC 98,000/WBC 25,000 (mixed)	Atypical—biopsy mandatory
Recurrence after drainage	Uncommon without ongoing source	150 mL re‐accumulated within 2 days	Red flag
MRI flow voids	Absent	Present—tumor angiogenesis	Red flag—suggests malignancy
Lesion wall (MRI)	Thin uniform rim	Thick, irregular, enhancing wall	Red flag
Response to antibiotics	Clinical improvement	No clinical improvement	Red flag if absent

Abbreviations: DDLPS, dedifferentiated liposarcoma; MRI, magnetic resonance imaging; RBC, red blood cells; WBC, white blood cells.

### 3.4 Comparison With Published Cases

Bayileyegn and Tareke [13] described a thigh DDLPS misidentified as a recurrent lipoma—illustrating clinical mimicry but without infective features or pre‐operative mortality. Djoumessi et al. [14] reported a massive thigh DDLPS extending to the retroperitoneum after 10 years of growth, treated with wide excision, with the patient surviving surgery. Suleiman et al. [15] documented a 4‐year giant WDL of the lateral thigh in a 46‐year‐old female, managed with wide local excision without pre‐operative fatality. Table 3 summarizes these comparisons.

**TABLE 3:** Comparison of the present case with selected published cases of extremity liposarcoma.

Study	Age/sex	Presentation	Imaging and diagnosis	Outcome
Bayileyegn and Tareke [13]	78M	Recurrent left thigh swelling; no fever or systemic symptoms	CT: heterogeneous mass; initially misidentified as lipoma; DDLPS on histology	Wide excision + radiotherapy; no pre‐operative death
Djoumessi et al. [14]	65F	Massive thigh mass for 10 years; painless; no infective features	CT/MRI: DDLPS extending to retroperitoneum; MDM2 positive	48 × 33 cm surgical specimen; patient survived
Suleiman et al. [15]	46F	4‐year giant painless lateral thigh mass; no systemic symptoms	Imaging: lipomatous mass; histology: WDL	Wide local excision; no pre‐operative death
Present case	60F	Fever, weight loss, refractory anemia; vascular mimicry → abscess mimicry; sterile purulent‐appearing aspirate	USG → hemangioma; CT → abscess; MRI → suspicious mass; Biopsy: DDLPS Grade 2; Gram stain/culture negative	Sudden death overnight post‐referral, pre‐operatively. First reported case with this triad and fatal pre‐operative outcome

Abbreviations: CT, computed tomography; DDLPS, dedifferentiated liposarcoma; MRI, magnetic resonance imaging; WDL, well‐differentiated liposarcoma.

### 3.5 Management

The standard of care for DDLPS is wide surgical excision with histologically negative (R0) margins, combined with adjuvant radiotherapy for local control [8]. Chemotherapy plays a limited role—anthracycline‐based regimens achieve objective responses in approximately 12% of advanced cases, with a median progression‐free survival of 4.6 months [16]. For the present patient, wide local excision had been planned following multidisciplinary evaluation. The pre‐operative fatal outcome precluded any curative or palliative intervention, underscoring the urgency of oncological management in advanced‐stage DDLPS.

### 3.6 Possible Mechanisms of Sudden Death

The cause of this patient's sudden overnight death cannot be formally established in the absence of autopsy. The following describes possible mechanisms supported by published evidence, rather than attributing a definitive cause.

- *Cancer‐associated venous thromboembolism with fatal pulmonary embolism* represents the most immediately life‐threatening plausible mechanism. In cancer patients, pulmonary embolism (PE) is the second leading cause of death after cancer progression, with a 4‐ to 7‐fold elevated VTE risk compared with the general population [17]. In‐hospital mortality from cancer‐associated PE is reported to range from 10% to 30% [18]. Autopsy‐based studies confirm PE as a frequently unrecognized direct cause of death in cancer patients with large compressive tumors of the lower extremity causing venous obstruction [19]. In this patient, unilateral leg oedema attributable to venous compression by the tumor may have reflected underlying deep vein thrombosis, with subsequent fatal PE as the terminal event—compounded by prolonged hospitalization, relative immobility, active malignancy, CKD, diabetes, and systemic inflammation [20].
- *Cancer cachexia‐related cardiac dysfunction* is a second plausible mechanism. The patient exhibited cardinal cachexia features: 6 kg involuntary weight loss within 1 month, anorexia, and refractory anemia. Tumor‐derived cytokines—particularly TNF‐alpha and IL‐6—drive progressive cardiac muscle atrophy, myocardial fibrosis, and diastolic and systolic dysfunction, potentially precipitating fatal arrhythmia or acute heart failure [21, 22, 23].
- *Acute tumor‐related hemodynamic compromise*—including intra‐tumoral hemorrhage, acute expansion of the necrotic cavity, or bacterial superinfection of liquefied necrotic contents with resultant septic shock—represents a third possible mechanism, given the enormous tumor burden (~19 × 12 cm on CT) and its proximity to pelvic vasculature.

## 4 Strength

This case highlights a rare presentation of extremity dedifferentiated liposarcoma with sequential vascular and abscess mimicry, including large‐volume sterile necrotic aspirate. It is supported by multimodal imaging and histopathology and underscores the clinical impact of delayed diagnosis through a fatal pre‐operative outcome.

## 5 Limitations

This case has several limitations. No autopsy was performed, so the exact cause of death remains unknown. MDM2/CDK4 testing was not done due to resource constraints, limiting diagnostic confirmation beyond classical histomorphology. As a single case, findings are not generalizable. Nevertheless, the comprehensive clinical, imaging, microbiological, and histopathological data provide valuable insight into atypical extremity DDLPS and its potential severity.

## 6 Conclusion

This case suggests that extremity DDLPS may present with a combination of vascular mimicry, systemic inflammatory syndrome, and large‐volume sterile necrotic aspirate that closely simulates pyogenic abscess—a diagnostic trap with potentially fatal consequences. Microbiologically sterile necrotic aspirate does not exclude malignancy; concurrent core biopsy of the lesion wall is essential in any deep soft tissue aspiration with atypical features. Clinicians should maintain a low threshold for malignancy in any progressively enlarging deep soft tissue mass, irrespective of radiological or inflammatory findings. Equally, patients with advanced sarcoma require proactive management of VTE risk, cachexia, and hemodynamic vulnerability from the point of diagnosis. Early biopsy is not merely diagnostically valuable; in cases such as this, it may be lifesaving.

## Author Contributions

**Rashid Shahriar Sazal:** conceptualization, writing – original draft, writing – review and editing, formal analysis, resources, supervision, project administration, data curation. **Md. Hashibul Hasan Shawon:** conceptualization, writing – review and editing, formal analysis, supervision, resources, visualization, methodology. **Mahin Zubaer:** conceptualization, writing – review and editing, formal analysis, supervision, investigation, funding acquisition, validation. **Md. Abul Kalam Azad:** writing – review and editing, investigation, project administration, formal analysis, supervision, resources, funding acquisition. **Khaled Mahbub Murshed:** writing – review and editing, formal analysis, software, resources, data curation, funding acquisition. **Kazi Ali Aftab:** formal analysis, investigation, funding acquisition, writing – review and editing, methodology, data curation, resources.

## Funding

The authors have nothing to report.

## Consent

Written informed consent for publication of clinical data and images was obtained from the patient's husband as the patient died. A copy of the signed consent form is available for review by the Editor‐in‐Chief upon request.

## Conflicts of Interest

The authors declare no conflicts of interest.

## Data Availability

The data that support the findings of this study are available from the corresponding author upon reasonable request.
